# Validation of epigenetic markers to identify colitis associated cancer: Results of module 1 of the ENDCAP-C study

**DOI:** 10.1016/j.ebiom.2018.11.034

**Published:** 2018-11-22

**Authors:** Andrew D. Beggs, Samir Mehta, Jonathan J. Deeks, Jonathan D. James, Germaine M. Caldwell, Mark P. Dilworth, Joanne D. Stockton, Daniel Blakeway, Valerie Pestinger, Alexandra Vince, Phillipe Taniere, Tariq Iqbal, Laura Magill, Glenn Matthews, Dion G. Morton, ENDCAP-C Module 1 Collaborative

**Affiliations:** a Institute of Cancer & Genomic Science, University of Birmingham, UK; b Birmingham Clinical Trials Unit, University of Birmingham, UK; c University Hospital Birmingham NHS Foundation Trust, UK; d National Institute for Health Research (NIHR), Birmingham Inflammation Biomedical Research Centre, UK

## Abstract

**Background:**

Chronic inflammation caused by ulcerative colitis (UC) causes a pro-neoplastic drive in the inflamed colon, leading to a markedly greater risk of invasive malignancy compared to the general population. Despite surveillance protocols, 50% of cases proceed to cancer before neoplasia is detected. The Enhanced Neoplasia Detection and Cancer Prevention in Chronic Colitis (ENDCaP-C) trial is an observational multi-centre test accuracy study to ascertain the role of molecular markers in improving the detection of dysplasia. We aimed to validate previously identified biomarkers of neoplasia in a retrospective cohort and create predictive models for later validation in a prospective cohort.

**Methods:**

A retrospective analysis using bisulphite pyrosequencing of an 11 marker panel (*SFRP1, SFRP2, SRP4, SRP5, WIF1, TUBB6, SOX7, APC1A, APC2, MINT1, RUNX3*) in samples from 35 patients with cancer, 78 with dysplasia and 343 without neoplasia undergoing surveillance for UC associated neoplasia across 6 medical centres. Predictive models for UC associated cancer/dysplasia were created in the setting of neoplastic and non-neoplastic mucosa.

**Findings:**

For neoplastic mucosa a five marker panel (*SFRP2, SFRP4, WIF1, APC1A, APC2*) was accurate in detecting pre-cancerous and invasive neoplasia (AUC = 0.83; 95% CI: 0.79, 0.88), and dysplasia (AUC = 0.88; (0.84, 0.91). For non-neoplastic mucosa a four marker panel (*APC1A, SFRP4, SFRP5, SOX7*) had modest accuracy (AUC = 0.68; 95% CI: 0.62,0.73) in predicting associated bowel neoplasia through the methylation signature of distant non-neoplastic colonic mucosa.

**Interpretation:**

This multiplex methylation marker panel is accurate in the detection of ulcerative colitis associated dysplasia and neoplasia and is currently being validated in a prospective clinical trial.

**Funding:**

The ENDCAP-C study was funded by the National Institute for Health Research Efficacy and Mechanism Evaluation (EME) Programme (11/100/29).

**Research in context**

**Evidence before this study**

• Chronic ulcerative colitis carries a substantially elevated risk of neoplasia as compared to an age matched unaffected population
• Current methods for detection of dysplasia and early neoplasia are unreliable
• Epigenetic markers have previously shown promise in the detection of UC associated dysplasia and neoplasia.

**Added value of this study**

• Methylation at a five marker panel (*SFRP2*, *SFRP4*, *WIF1*, *APC1A*, *APC2*) panel highly accurately detects ulcerative colitis associated dysplasia and cancer in a retrospective cohort from patients treated at 6 different hospitals in the UK
• A second methylation panel ((*APC1A, SFRP4, SFRP5, SOX7*) may have value as an adjunct to colonoscopy in identifying high risk patients by providing predictive markers in random biopsies of background mucosa.

**Implications of all the available evidence**

• A multi-marker methylation panel may be able to identify patients with high risk dysplasia

Alt-text: Unlabelled Box

## 1 Introduction

Chronic inflammation caused by Ulcerative Colitis (UC) causes a pro-neoplastic drive in the inflamed colon, leading to a markedly greater risk of invasive malignancy compared to the general population [1]. Although rates of UC associated neoplasia seem to be decreasing [2], due in part to improved medical control of inflammation, there remains a significant risk beyond the background risk of colorectal cancer (CRC). The risk is particularly pronounced in patients with extensive colitis and an inflammatory bowel disease (IBD) diagnosis before 30 years of age [3].

Despite colonoscopic surveillance protocols [4], 50% of cases are reported to have developed invasive cancer before neoplasia is detected. The disease is frequently multifocal, presumed due to the diffuse sensitisation of the large bowel mucosa by the chronic inflammatory process. Mutational events, such as *KRAS* and *TP53* mutation [5] have been observed as part of this field cancerization effect in UC, however no consistent pattern has been demonstrated.

Endoscopic therapy can provide local control of early dysplastic lesions, but enhanced detection strategies are required to aid early detection and ensure that progressive dysplasia is not missed during surveillance [[6], [7], [8]].

Chronic inflammation has been demonstrated to promote aberrant DNA methylation in conditions such as ulcerative colitis [9]. This may be due to a direct chemical effect causing cytosine methylation in the inflamed colon. Use of abnormal DNA methylation as a biomarker for ulcerative colitis associated neoplasia has considerable theoretical advantages; firstly methylation tends to be gene-centric [10], centering around CpG islands and secondly it is usually homogenously distributed within the CpG island, having a functional effect on transcription factor binding and thus gene expression. This homogeneity facilitates simpler detection of abnormal methylation patterns. Another advantageous property of assaying methylation is that it tends to occur as part of a “field cancerization” effect [11] whereby associated changes in methylation extend out past the dysplastic lesion in the colon and thus can be detected in apparently normal mucosa some distance from the lesion [12,13].

It has been demonstrated that hypermethylation of members of the Frizzled pathway, involved in Wnt signalling regulation, are associated with colorectal tumouriogenesis [[14], [15], [16]]. Dhir et al. [17] carried out an analysis of methylation of Wnt signalling genes (*APC1A, APC2, SFRP1, SFRP2, SFRP4, SFRP5, DKK1, DKK3, WIF1* and *LKB1*) in the development of UC associated neoplasia, finding that methylation of *SFRP1/2* and *APC1A/2* were associated with the progression to invasive disease. Guo et al. [18] demonstrated that SOX7, an independent checkpoint for beta-catenin function can be hypermethylated in colorectal cancer and may play a role in UC associated neoplasia.

Genetic variation in *RUNX3* has been demonstrated as a risk factor for the development of ulcerative colitis [19] and Garritty-Park et al. [20] demonstrated that hypermethylation of *RUNX3*, and *MINT1* could be detected in the non-neoplastic mucosa from patients with colitis associated neoplasia. In our own analysis of colitis associated mucosa [21] utilising the Illumina Methylation450 platform, we identified an association between hypomethylation of *TUBB6* in non-neoplastic colonic mucosa from patients with UC associated neoplasia.

In the Enhanced Neoplasia Detection and Cancer Prevention in Chronic Colitis (ENDCaP-C) study, we set out to establish whether an optimised methylation marker panel of suitable specificity could improve detection of early neoplastic lesions at colonoscopic surveillance diagnostic accuracy. This initial phase (Module 1) aims to measure the accuracy of an optimised panel of markers on a multicentre, retrospective cohort of patients with ulcerative colitis before assessing their utility in a prospective multi-centre test accuracy study (Module 3).

We aimed to:

1) Establish and optimise a multi-marker methylation panel for the detection of colitis associated neoplasia.
2) Measure the accuracy of this panel in a retrospective multicentre cohort of patients with colitis associated neoplasia.

## 2 Materials & methods

### 2.1 Patient recruitment

**Fig. 1:**
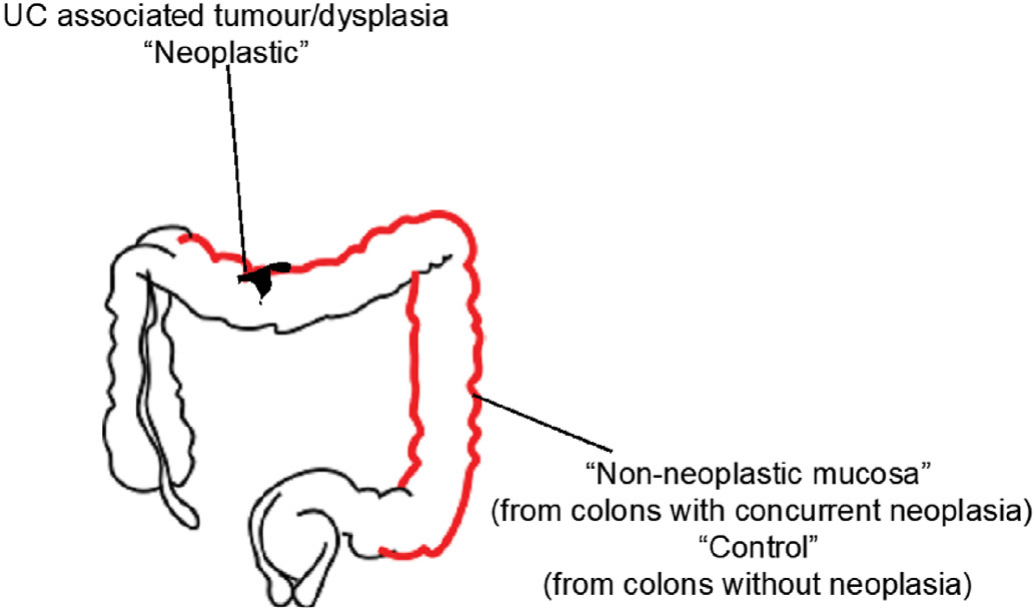
Diagram of colonoscopic sampling from patients for ENDCAP-C study.

Patients were identified from archived histology biopsy samples in 6 hospitals across the West Midlands area. Patients were identified through tracing endoscopy records and correlation with histology reports. Searches were restricted to endoscopies after January 1996, because of changes to formalin fixation at that time, and *before* January 2014 to minimise missed neoplasia through identification during the follow up period. Mucosal biopsies were classified via pathological examination using H&E sections as either *neoplasia*, defined as any of adenocarcinoma, high-grade or low-grade dysplasia; *matched non-neoplastic* defined as non- neoplastic chronically inflamed colonic mucosal biopsies taken distant from areas of neoplasia as distant as possible from the original neoplastic region (see Fig. 1); *control* defined as colonic mucosa, sampled from patients with chronic ulcerative colitis of duration >8 years and extending to the splenic flexure or beyond OR patients with a diagnosis of both UC and PSC who had been screened for neoplasia without it being found and in whom no neoplasia was seen in follow up after the biopsy was taken. Ethical approval was from South Birmingham Research Ethics Committee (Ref: 08/H1207/104).

### 2.2 Sample processing

Biopsy samples from identified patients were retrieved from the histopathology archives at the 6 collaborating hospitals. For those patients with neoplasia in the large bowel, separate biopsies from different colonic segments were selected alongside the neoplastic biopsy. All blocks underwent central histological review by PT, with representative sections undergoing DNA extraction. Dysplasia was defined as any of altered nuclear/cytoplasmic ratio, increased cell size and/or an increase in mitotic figures. DNA extraction of neoplasia was performed by needle macrodissection to enrich for tumour material, and macrodissected material was extracted using the FFPE protocol of the Qiagen DNEasy FFPE kit (Qiagen, UK). Extracted DNA was quantified by both Nanodrop spectrophotometry and Qubit fluorimetry.

Neoplasia, matched non-neoplastic and control samples were included in mixed batches to ensure that test performance could be analysed at sequential analyses across the study duration. Each sample was labelled with only a study sample identification number and assays undertaken blinded to neoplasia status.

### 2.3 DNA methylation analysis

500 ng of extracted DNA was bisulphite converted using a Zymo EZ-DNA methylation bisulphite conversion kit according to the manufacturer's protocol. Two microliters of eluted bisulphite converted DNA was utilised in a pyrosequencing PCR reaction using the Qiagen PyroMark PCR kit according to the manufacturer's protocol in a 25uL reaction volume. PCR products were run on a 2% agarose gel with a DNA size ladder and successful amplification was defined as the presence of a band at the appropriate size for the marker run. PCR products were cleaned using streptavidin beads, washed and mixed with the requisite pyrosequencing primers. These were then sequenced on a Qiagen PyroMark Q96 instrument. All reactions were run with 100% methylated and unmethylated DNA positive and negative controls, as well as a water reaction. Methylated DNA was generated by incubating 1μg of blood derived control DNA with M.Ssi CpG methyltransferase (New England Biolabs). Unmethylated DNA was generated by whole genome amplification of 10 ng of blood derived control DNA using the Qiagen Repli-G Mini kit.

The marker panel chosen for this experiment was based on previous findings and consisted of the following markers: *SFRP1*, *SFRP2*, *SFRP4*, *SFRP5*, *WIF1*, *TUBB6*, *SOX7*, *APC1A*, *APC2*, *MINT1*, *RUNX3*. Primer sequences, chromosomal positions and reaction conditions are shown in Supplementary Table 1. After each run, sample data was examined using Qiagen PyroMark Q96 software. Samples that had failed Qiagen quality metrics were marked as failed on the sample sheet.

### 2.4 Sample size

The original planned sample size of 160 neoplastic and 320 control samples was determined to provide adequate events to robustly develop a model (with >10 events per marker) and provide estimates of sensitivity and specificity with adequate precision (with 95% confidence interval width <16% for sensitivity and <12% for specificity). The 2:1 sampling ratio is determined based on access to sample banks.

### 2.5 Statistical analysis

Where biomarkers were examined at multiple CpG sites the mean CpG value across sites was used in all analyses for consistency and as methylation within small regions tends to be distributed homogenously [10]. Statistical analysis was then undertaken in three steps.

First, results for each batch of samples for each of the 11 biomarkers (*SFRP1*, *SFRP2*, *SFRP4*, *SFRP5*, *WIF1*, *TUBB6*, *SOX7*, *APC1A*, *APC2*, *MINT1*, *RUNX3*.) were evaluated in a group sequential analysis following the O'Brien and Fleming (OBF) method [22], to assess whether further testing of each biomarker was justified or considered futile. Sequential boundaries were constructed according to the OBF method, the t-statistic computed for each biomarker at each analysis step, and comparison made to the predefined boundary values to test for statistical significance or futility (see Supplementary Fig. 1).

In this first stage the distributions of the biomarker values were unknown, and data were analysed without transformation. Once all data were accumulated, visual inspection of histograms demonstrated positive skew (Supplementary Fig. 2), and the mean biomarker values for each sample were log-transformed prior to further analysis.

In the second stage markers with responses suitable for inclusion in the predictive models were selected. The ability of each marker to discriminate was described by computing the ratio of geometric means (with 95% confidence interval) and statistical significance was assessed by two-sample *t*-tests undertaken on the log transformed scale. Comparisons were made [1] between neoplasia samples and control samples, and [2] between matched non-neoplastic samples and control samples. Biomarkers which showed significant discrimination (p < 0.05) and with amplification rates >85% were included in the predictive model.

In stage 3 predictive models were fitted using logistic regression with outcome (1 = Sample or 0 = Control) and the mean log CpG value for each patient for the biomarkers selected in Stage 2. Only samples which had complete data for the selected biomarkers were initially included in these analyses. Three separate models were constructed: [1] differentiating neoplasia samples from control samples; [2] differentiating dysplasia samples from control samples; and [3] differentiating matched non-neoplastic samples from control samples. Model 2 used the same patients and biomarker selection as Model 1, but excluded any samples which were classed as adenocarcinomas.

Discriminatory performance for each model was measured by the area under the ROC curve (AUC). We estimated optimism by fitting the predicted model in 100 bootstrap samples and computing the average difference between the AUC in the bootstrap samples and in the original data. We applied the computed shrinkage factor to the parameter estimates [23]. The final models were produced including all samples, using multiple imputation using chained equations to impute missing biomarker data. Multiple imputation models used 50 iterations with pathology categorisation, sample type and measurements of all other biomarkers as predictors. The model coefficients were corrected for optimism by application of the shrinkage factor. To facilitate application of the model when individual or pairs of biomarkers are unavailable, reduced models were computed omitting each biomarker and possible pair of biomarkers from the multiple imputation model.

One biomarker (TUBB6) was not selected for inclusion in Models 1 and 2 in the stage 1 OBF analysis on untransformed data, but did show significant differences in stage 2 once log transformations had been applied. Models 1 and 2 were fitted with and without this biomarker.

The clinical team considered that a positive test result should have a positive predictive value of at least 20% to be of clinical value. Given an assumed background incidence of 4% this corresponds to the point on the ROC curve with a positive likelihood ratio (sensitivity/(1-specificity)) of 6. The threshold at this point was identified from the ROC tabulation of each predictive equation, and estimates of sensitivity and specificity obtained. We also identified thresholds for each model which corresponds with 90% of cases being detected.

## 3 Results

In total, 838 blocks from 575 patients were collected from 6 participating hospitals. Of these, 269 blocks were not used in the study because they were duplicates from the same patient, or deemed not useable after histological review. This left 569 blocks from 456 patients undergoing surveillance, consisting of 113 neoplastic, 113 matched non-neoplastic and 343 control blocks (Table 1). Of the neoplastic biopsy samples, 35/113 contained adenocarcinoma and the remaining 78/113 harboured dysplasia only. Baseline data for participants providing these blocks is shown in Table 2.

**Table 1:** Samples graded according to histology and inflammation (central assessments).

Histopathological type	Patients with neoplasia(n = 113)		Patients without neoplasia(control) (n = 343)	Total (n = 569)
	Neoplastic samples	Matched non-neoplastic samples		
Number of blocks mean (sd)	2.9 (4.4)		1.2 (0.5)	1.6 (2.3)
Median [IQR]	2 [2–2]		1 [1–1]	1 [1–2]
Range	2–42		1–6	1–42
Adenocarcinoma	35 (31%)	–	–	35 (6%)
High grade dysplasia	4 (4%)	–	–	4 (1%)
Low grade dysplasia	74 (65%)	–	–	74 (13%)
Active chronic inflammation	–	33 (29%)	83 (25%)	116 (20%)
Non active chronic inflammation	–	69 (61%)	204 (59%)	273 (48%)
Normal mucosa	–	11 (10%)	56 (16%)	67 (12%)

**Table 2:** Baseline patient data.

Baseline characteristics		Patients with neoplasian (%age)	Patients without neoplasia(control) n (%age)	Total n (%age)
		(n = 113)	(n = 343)	(N = 456)
Montreal classification	Distal (Recto-Sigmoid)	23 (20%)	56 (16%)	79 (17%)
	Left-sided (to splenic flexure)	17 (15%)	61 (18%)	78 (17%)
	Extensive (beyond splenic flexure)	67 (59%)	203 (59%)	270 (59%)
	Unknown/Missing	6 (5%)	23 (7%)	29 (6%)
Smoker	No	75 (66%)	197 (57%)	272 (60%)
	Yes	7 (6%)	9 (2%)	16 (4%)
	Unknown	22 (19%)	125 (36%)	147 (32%)
	Ex-smoker	9 (8%)	12 (3%)	21 (5%)
Primary sclerosing cholangitis	No	100 (88%)	293 (85%)	393 (86%)
	Yes	8 (7%)	46 (13%)	54 (12%)
	Unknown/Missing	5 (4%)	(1%)	9 (2%)
Family history of inflammatory bowel disease	No	85 (75%)	249 (73%)	334 (73%)
	Yes	4 (4%)	20 (6%)	24 (5%)
	Unknown/Missing	24 (21%)	74 (22%)	98 (21%)
Family history of colorectal cancer	No	83 (73%)	257 (75%)	340 (75%)
	Yes	6 (5%)	6 (2%)	12 (3%)
	Unknown	17 (15%)	13 (4%)	30 (7%)
	Missing	7 (6%)	67 (20%)	74 (16%)

### 3.1 Selection of biomarkers

Eight of the eleven methylation markers had an amplification success rate of >85% (Table 3 and Supplementary Table 2). The three remaining primer sets were within the promotor regions of *SFRP1*, MINT1 and RUNX3. Because of the reduced reliability, these 3 were not taken forward to further analysis.

**Table 3:** Distribution and comparison of methylation markers by sample type.

Biomarker	Geometric mean (95% Confidence interval)			Ratio of geometric means(95% confidence interval); P-Value^a^	
	Neoplastic (n = 113)	Matched non-neoplastic(n = 113)	Control (n = 343)	Neoplastic vs. control	Non-neoplasticvs. control
sFRP2	22.1 (19.7, 24.9)	14.0 (12.8, 15.4)	14.1 (13.4, 14.9)	1.57 (1.40, 1.76)	0.99 (0.89, 1.11)
	(n = 105)	(n = 106)	(n = 303)	P < 0.0001	P = 0.92
sFRP4	44.7 (42.1, 47.5)	34.4 (31.8, 37.2)	32.0 (31.0, 33.1)	1.40 (1.31, 1.49)	1.07 (1.00, 1.15)
	(n = 108)	(n = 109)	(n = 312)	P < 0.0001	P = 0.057
WIF1	21.6 (18.6, 25.2)	12.8 (11.1, 14.8)	13.9 (12.8, 15.0)	1.56 (1.33, 1.83)	0.93 (0.79, 1.08)
	(n = 104)	(n = 105)	(n = 292)	P < 0.0001	P = 0.33
APC1A	2.92 (2.37, 3.60)	2.54 (2.16, 3.00)	1.99 (1.83, 2.17)	1.47 (1.22, 1.77)	1.28 (1.07, 1.52)
	(n = 102)	(n = 102)	(n = 297)	P = 0.0001	P = 0.006
APC2	35.4 (32.1, 39.0)	22.3 (20.4, 24.4)	20.2 (18.9, 21.5)	1.76 (1.55, 1.99)	1.11 (0.98, 1.26)
	(n = 111)	(n = 106)	(n = 322)	P < 0.0001	P = 0.12
sFRP1	35.7 (30.5, 41.9)	24.1 (21.7, 26.7)	25.1 (23.2, 27.1)	1.42 (1.21, 1.67)	0.96 (0.82, 1.13)
	(n = 39)	(n = 29)	(n = 118)	P < 0.0001	P = 0.62
sFRP5	7.14 (5.75, 8.87)	4.90 (4.08, 5.90)	6.40 (5.64, 7.27)	1.12 (0.87, 1.43)	0.77 (0.60, 0.98)
	(n = 102)	(n = 95)	(n = 275)	P = 0.38	P = 0.03
MINT1	4.14 (3.32, 5.16)	3.40 (2.87, 4.04)	3.13 (2.82, 3.48)	1.32 (1.06, 1.64)	1.09 (0.89, 1.33)
	(n = 73)	(n = 70)	(n = 200)	P = 0.012	P = 0.42
RUNX3	8.73 (7.15, 10.7)	7.58 (6.37, 9.02)	7.44 (6.68, 8.29)	1.17 (0.94, 1.46)	1.02 (0.83, 1.25)
	(n = 87)	(n = 97)	(n = 248)	P = 0.15	P = 0.86
SOX7	5.70 (4.60, 7.06)	3.92 (3.41, 4.51)	5.41 (4.88, 5.99)	1.05 (0.85, 1.30)	0.73 (0.60, 0.87)
	(n = 100)	(n = 106)	(n = 280)	P = 0.63	P = 0.001
TUBB6	12.2 (10.5, 14.2)	8.04 (6.93, 9.34)	9.34 (8.52, 10.23)	1.31 (1.10, 1.56)	0.86 (0.72, 1.03)
	(n = 108)	(n = 95)	(n = 292)	P = 0.003	P = 0.11

a Computed from a 2-sample t-test on log transformed data.

In the stage 2 analysis, five markers accurately discriminated between neoplasia and control samples with p < 0.0001 – *SFRP2*, *SFRP4*, *WIF1*, *APC1A* and *APC2* (Table 3); with between 40% and 76% increases in geometric mean values. *TUBB6* showed a smaller (31%) increase but which was also strongly significant (p = 0.003).

Comparison in samples of methylation in the background mucosa (from patients with colitis associated neoplasia) with control patients (with chronic UC only) showed some discrimination in four of the eight promotor regions. *SFRP4*, *APC1A*, *SFRP5* and *SOX7* (Table 3). Two of these markers *SFRP4* and *APC1A* showed increases of 7% and 28% respectively, in geometric mean values; the other two, *SFRP5* and *SOX7* showed decreases of 23% and 27% respectively.

### 3.2 Performance of predictive models

**Fig. 2:**
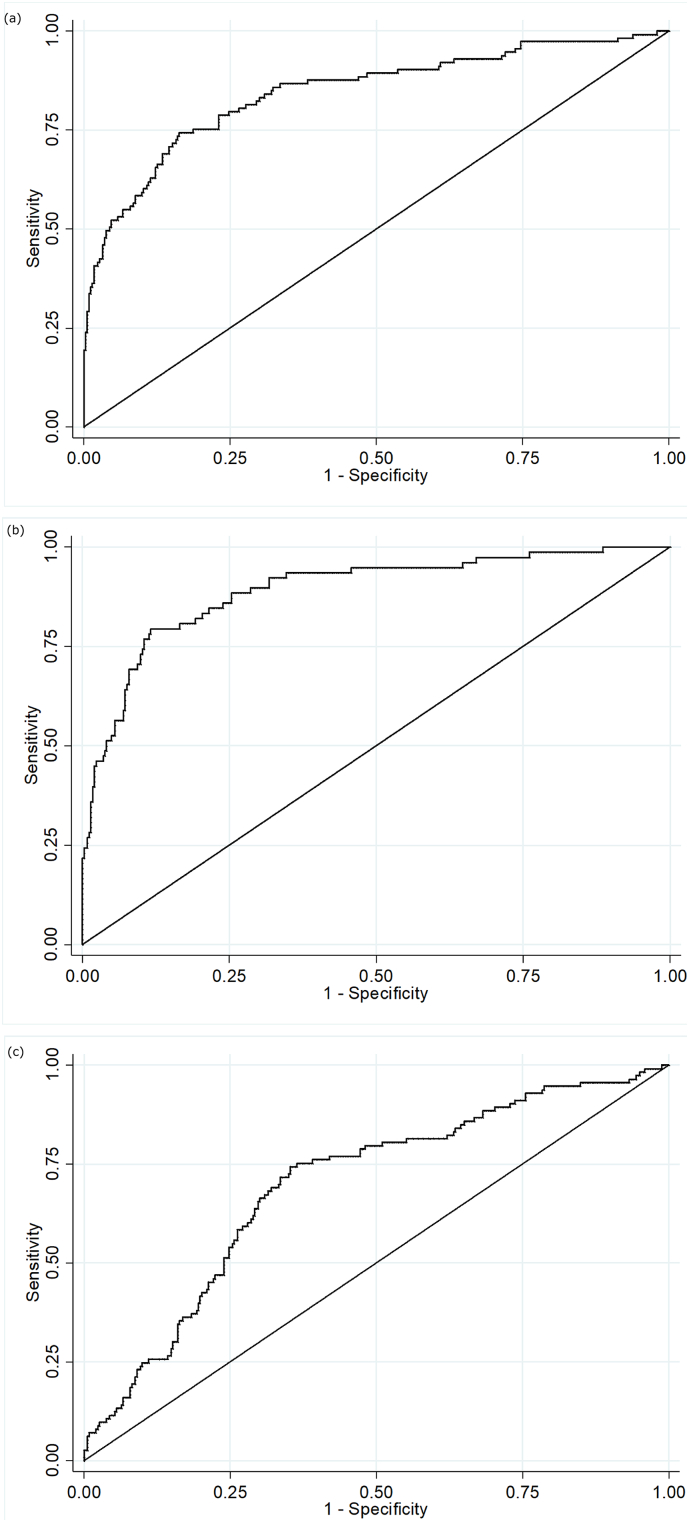
ROC curves for final fitted predictive models (after multiple imputation). A = Model 1 Neoplasia vs control; B = Model 2 Dysplasia vs control; C = Model 3 Matched non-neoplastic vs control. X-axis = 1-specificity; Y-axis = Sensitivity.

Predictive models to discriminate neoplasia samples from controls had good discrimination (Table 4). The optimism adjusted AUC for Model 1 detecting all neoplasia was 0.86 (95% CI 0.81, 0.91) for the complete case analysis (with a shrinkage factor of 0.93) but lower at 0.83 (95% CI 0.79, 0.88) for the model using multiple imputation (Fig. 2A). Addition of *TUBB6* only increased the AUC by 0.001. When considered together in the panel, all markers other than *WIF1* showed significant independent predictive value.

**Table 4:** Estimates of discrimination, optimism and shrinkage for fitted models.

Model^a^	Optimism^b^	Shrinkage^b^	Complete caseAUC (95% CI)	Complete case adjusted foroptimism AUC (95% CI)	Multiple imputationAUC (95% CI)	Multiple imputation adjustedfor optimism AUC (95% CI)
Model 1	0.012	0.93	0.871 (0.822, 0.919)	0.859 (0.810, 0.907)	0.845 (0.799, 0.891)	0.833 (0.787, 0.879)
with TUBB6	0.015	0.91	0.875 (0.826, 0.923)	0.860 (0.811, 0.908)	0.848 (0.802, 0.894)	0.833 (0.787, 0.879)
Model 2	0.012	0.91	0.930 (0.892, 0.967)	0.918 (0.880, 0.955)	0.892 (0.849, 0.934)	0.880 (0.837, 0.922)
with TUBB6	0.015	0.88	0.932 (0.894, 0.970)	0.917 (0.879, 0.955)	0.894 (0.852, 0.937)	0.879 (0.837, 0.922)
Model 3	0.021	0.90	0.682 (0.614, 0.750)	0.661 (0.593, 0.729)	0.696 (0.640, 0.751)	0.675 (0.619, 0.730)

a Model 1 compared neoplasia with control; Model 2 compared dysplasia with control; Model 3 compared matched non-neoplastic with control.

b Optimism and shrinkage were estimated from internal validation using bootstrap sampling.

Discrimination of Model 2 predicting only dysplasia (excluding adenocarcinoma cases) was higher with an optimism corrected AUC of 0.92 (95% CI 0.88, 0.96) for the analysis of complete cases (with a shrinkage factor of 0.91), and 0.88 (95% CI 0.84, 0.92) for the model using multiple imputation (Fig. 2B). Again adding *TUBB6* made little difference, decreasing the AUC by 0.001. We report coefficients for Model 1 based on the multiple imputation dataset in Supplementary Table 3A and for Model 2 in Supplementary Table 3B. When considered together in the panel, all markers other than *WIF1* showed significant independent predictive value.

The predictive model to discriminate samples where there is methylation in the background mucosa (the matched non-neoplastic samples, model 3) from controls had poorer discrimination, with an optimism adjusted AUC of 0.66 (95% CI 0.59, 0.73) for the complete case model (shrinkage factor 0.91), and 0.68 (95% CI 0.62, 0.73) for the model using multiple imputation (Fig. 2C). We report coefficients for Model 3 based on the multiple imputation dataset in Supplementary Table 3C. For *SFRP5* and *SOX7* lower levels of methylation were associated with neoplastic change in background mucosa. When considered together only *APC1A* and *SOX7* showed significant independent predictive value.

The calibration plot for Model 1 after multiple imputation suggested that our final model for neoplasia detection after multiple imputation was reasonably well calibrated, with slight overestimation of probability at lower risk and overestimation at higher risk (Supplementary Fig. 3).

### 3.3 Identification of diagnostic threshold

We identified the value of the predictive model corresponding to a likelihood ratio of at least 6, to identify thresholds which would have a positive predictive value of at least 20% when disease prevalence was 4%. This corresponded to a threshold of 0.40 for Model 1, 0.28 for Model 2, and 0.50 for Model 3. Sensitivity and specificity (with 95% confidence intervals) at this threshold were 58.4% (48.8%, 67.6%) and 90.38% (86.8%, 93.3%) for Model 1, 79.5% (71.0%, 86.6%) and 86.9% (82.8%, 90.3%) for Model 2 and 7.1% (3.1%, 13.5%) and 98.8% (97.0%, 99.7%) for Model 3.

To achieve a sensitivity of at least 90%: Model 1 would use a threshold of 0.11 with a specificity of 46.4% (41.1%, 51.8%) and positive predictive value of 6.6% (5.9%, 7.3%); Model 2 would use a threshold of 0.11 with a specificity of 68.2% (63.0%, 73.1%) and positive predictive value of 10.7% (9.11%, 12.3%); and Model 3 would use a threshold of 0.19 with a specificity of 27.1% (22.5%, 32.1%) and positive predictive value of 4.9% (4.5%, 5.3%).

### 3.4 Models for missing data

For Model 1, to provide models for scenarios where data on less than five of the chosen biomarkers amplify, separate models accounting for all possible scenarios of at least three biomarkers were created through re-analysis of the multiple imputation model with reduced sets of predictor variables. This created an additional 15 models, the coefficients for which are reported in Supplementary Table 4.

## 4 Discussion

In this study we have demonstrated that a five marker methylation marker panel accurately predicts ulcerative colitis associated dysplasia and invasive neoplasia from formalin fixed mucosal biopsies taken at endoscopy, both dysplastic and “normal” mucosa within the potential field of epigenetic change. The generalisability of these findings is increased through evaluation across a diverse population. The study has also identified a second marker panel found in the background mucosa that is (more weakly) associated with neoplastic change. Our panels utilise epigenetic biomarkers, which are emerging as a reproducible method of quantifying disease risk in a population and suggest that these markers may add value to endoscopic detection of colitis associated neoplasia.

Specific patterns of methylation change have been observed in colorectal cancer [24] as well as specific changes observed in the transition from dysplastic colorectal adenoma to malignant adenocarcinoma [25] suggesting that methylation has good sensitivity as a biomarker of disease. To our knowledge, this is the first multiplex methylation biomarker panel in colorectal cancer.

Other cancer types have demonstrated potential utility of methylation analysis in screening for invasive disease. The UroMark study [26] investigated the utility of a multiplex bisulphite PCR amplicon next generation sequencing in the detection of muscle invasive bladder cancer in voided urine. Using a 150 marker panel based on differentially methylated CpG loci in a discovery study, they validated their marker panel in a cohort of 274 patients with muscle invasive bladder cancer, finding an overall AUC = 0.97. A significant advantage of their cohort was the ability to develop a marker panel based on a large sample number epigenome wide association study, an approach that might be appropriate in UC associated dysplasia, which has a heterogeneous genetic profile.

Generally it is accepted that AUC > 0.80 represents a “good” biomarker panel for the detection of disease and multiple marker panels using several different technologies have been developed across multiple disease types that have reached this target [[27], [28], [29]]. The AUC for detection of colitis associated neoplasia suggests this is a reliable test for neoplasia, however we are seeking to enhance detection, not replace colonoscopy. Ultimately it is the early detection of occult disease that will determine the value of this assay, and that requires prospective evaluation, which is already underway.

It is likely with more extensive epigenetic analysis of this retrospective cohort we could enhance our observed AUC level. The study has established that a reliable and robust assay can be developed for these patients. We are currently developing an NGS based multiplex assay to increase clinical utility. Whilst we under recruited tumour blocks (113 rather than the planned 160) this still provided over 20 events per variable for generating our 5-marker models, but will have increased the maximum 95% confidence interval width for sensitivity by 3%. The study was however carried out on a genetically and geographically diverse population, supporting the generalisability of our findings.

This study has also developed a novel marker set for predicting the presence of co-existing neoplasia from analysis of the background mucosa. Unsurprisingly, the AUC value for this is significantly lower, and the test therefor less robust. But the impact of these markers can only be determined in a longitudinal analysis. These methylation changes are present in a subset of the UC population, without associated neoplasia. Follow up of these patients will be required to determine whether this represents a high risk population for whom therapeutic cancer prevention strategies can be developed.

Three markers (*SFRP1*, *MINT1* and *RUNX3*) were not taken forward due to poor amplification rates during PCR, which we hypothesised was because of the high GC content of these regions making primer design difficult across FFPE derived DNA. Also, *WIF1* methylation was found not to contribute to the disease model, presumably because because its methylation levels were similar to other genes that were analysed within this study that are all modulators of the Wnt signalling pathway. We also noticed that the direction of methylation (towards hypomethylation) differing for the markers in model 3, we hypothesise that this is because of the previously demonstrated “wave” of hypomethylation [30] that occurs as a precursor to invasive malignancy and therefore should occur in the disease associated non-dysplastic mucosa we sampled here.

This study has proposed a methylation marker panel developed from analysed a retrospective multicentre cohort of ulcerative colitis patients with and without colitis associated dysplasia. We are now completing a prospective diagnostic accuracy study, of 820 UC patients within a surveillance programme, to evaluate the diagnostic utility of this panel test.

In conclusion, we have successfully developed a multiplex methylation marker panel for the detection of ulcerative colitis associated dysplasia and neoplasia which has validated in a retrospective cohort and is currently being evaluated in the context of a prospective clinical trial.

## Appendix A Supplementary data

Supplementary material Image 1
