# Supplementary material for: Validation of epigenetic markers to identify colitis associated cancer: Results of module 1 of the ENDCAP-C study
Source: eBioMedicine. 2018 Nov 22;39:265–71. doi: 10.1016/j.ebiom.2018.11.034 (PMC6355942; doi:10.1016/j.ebiom.2018.11.034)
Supplement: Supplementary file 1 — Supplementary material [file mmc1.docx]

**SUPPLEMENTARY TABLES**

**Supplementary Table 1:**

**Primer sequences, chromosomal positions and reaction conditions from chosen 11 marker panel. (Key Btn – Biotin)**

| Marker | Chromosomal position | Forward primer | Reverse primer | Sequencing primer | Annealing temperature |
| --- | --- | --- | --- | --- | --- |
| SFRP1 | chr12:65,444,407-65,515,116 | GGGTGTTTTTGTTTAATAAGAATTGT | ACTTAAACATCTCCAACCAATAAAAACC | GTTGAGGGAGTTGTAG | 56C |
| SFRP2 | Chr 4, BP 1547103XX-1547103XX | Qiagen assay ID PM00018809 |  |  | 56C |
| SFRP4 | Chr 7, BP 379558XX-379558XX | Qiagen assay ID  PM00029834 |  |  | 56C |
| SFRP5 | Chr 10, BP 995313XX-995314XX | Qiagen assay ID PM00147182 |  |  | 56C |
| WIF1 | chr12:65,444,407-65,515,116 | GTTTTGGTTGAGGGAGTTG | CCAACAAACACAAAAAAATACTCCA | GTTGAGGGAGTTGTAG | 56C |
| TUBB6 | chr18:12307624-12307734 | AGGAGTAGGTTGTATAGAT | Btn-TCTCCCAAAATACAAAAACCATTCCTCT | ATTAGGAGTAGGAGGTGTTTATT | 48C |
| SOX7 |  | Qiagen assay ID PM00138229 |  |  | 56C |
| APC1A | chr5:112072610-112073685 | GGGGTTAGGGTTAGGTAGG | TCCAACCAATTACACAACTACTTCTCTCT | AGGGTTAGGTAGGTT | 56C |
| APC2 | chr19:1449557-1450887 | AGTTTGTAGTGGGAGAGT | CTACCTACCTCCAACTCAAATAACAAC | GTTTGTAGTGGGAGAGTTA | 56C |
| MINT1 | chr5:75378746-75381277 | GTGTTGAGAGAGTTTGAAAGAAATATTAT | CCCAAAAAATTTTTACTAAATAAAAAC | GAATTTTTAAATTTTTTTATATATA | 56C |
| RUNX3 | chr1:25254900-25258752 | GGGAGTTAGGGGTAAATGTTAGAAAT | CCCCCAACCCCAAATTACAAAAATCACA | GGTAAATGTTAGAAATTTGTTTAGA | 56C |

**Supplementary Table 2:**

**Amplification data for each biomarker**

| **Biomarker** | **Amplification status** | **Neoplastic**  **(N=113)** | **Non-Neoplastic (N=113)** | **All Controls**  **(N=343)** | **Total**  **(N=569)** |
| --- | --- | --- | --- | --- | --- |
| **SFRP1** | Not amplified | 72 (63.7%) | 84 (74.3%) | 223 (65%) | 379 (66.6%) |
|  | Amplified | 39 (34.5%) | 29 (25.7%) | 119 (34.7%) | 187 (32.9%) |
|  | Not done | 2 (1.8%) | 0 (0%) | 1 (.3%) | 3 (.5%) |
| **SFRP2** | Not amplified | 8 (7.1%) | 7 (6.2%) | 38 (11.1%) | 53 (9.3%) |
|  | Amplified | 105 (92.9%) | 106 (93.8%) | 305 (88.9%) | 516 (90.7%) |
|  | Not done | 0 (0%) | 0 (0%) | 0 (0%) | 0 (0%) |
| **SFRP4** | Not amplified | 5 (4.4%) | 4 (3.5%) | 30 (8.7%) | 39 (6.9%) |
|  | Amplified | 108 (95.6%) | 109 (96.5%) | 313 (91.3%) | 530 (93.1%) |
|  | Not done | 0 (0%) | 0 (0%) | 0 (0%) | 0 (0%) |
| **SFRP5** | Not amplified | 3 (2.7%) | 1 (.9%) | 16 (4.7%) | 20 (3.5%) |
|  | Amplified | 110 (97.3%) | 112 (99.1%) | 323 (94.2%) | 545 (95.8%) |
|  | Not done | 0 (0%) | 0 (0%) | 4 (1.2%) | 4 (.7%) |
| **WIF1** | Not amplified | 7 (6.2%) | 8 (7.1%) | 45 (13.1%) | 60 (10.5%) |
|  | Amplified | 106 (93.8%) | 105 (92.9%) | 294 (85.7%) | 505 (88.8%) |
|  | Not done | 0 (0%) | 0 (0%) | 4 (1.2%) | 4 (.7%) |
| **TUBB6** | Not amplified | 3 (2.7%) | 9 (8%) | 23 (6.7%) | 35 (6.2%) |
|  | Amplified | 110 (97.3%) | 104 (92%) | 316 (92.1%) | 530 (93.1%) |
|  | Not done | 0 (0%) | 0 (0%) | 4 (1.2%) | 4 (.7%) |
| **SOX7** | Not amplified | 9 (8%) | 1 (.9%) | 47 (13.7%) | 57 (10%) |
|  | Amplified | 104 (92%) | 112 (99.1%) | 292 (85.1%) | 508 (89.3%) |
|  | Not done | 0 (0%) | 0 (0%) | 4 (1.2%) | 4 (.7%) |
| **APC1A** | Not amplified | 2 (1.8%) | 5 (4.4%) | 20 (5.8%) | 27 (4.7%) |
|  | Amplified | 111 (98.2%) | 108 (95.6%) | 319 (93%) | 538 (94.6%) |
|  | Not done | 0 (0%) | 0 (0%) | 4 (1.2%) | 4 (.7%) |
| **APC2** | Not amplified | 2 (1.8%) | 7 (6.2%) | 17 (5%) | 26 (4.6%) |
|  | Amplified | 111 (98.2%) | 106 (93.8%) | 322 (93.9%) | 539 (94.7%) |
|  | Not done | 0 (0%) | 0 (0%) | 4 (1.2%) | 4 (.7%) |
| **MINT1** | Not amplified | 33 (29.2%) | 26 (23%) | 102 (29.7%) | 161 (28.3%) |
|  | Amplified | 80 (70.8%) | 87 (77%) | 238 (69.4%) | 405 (71.2%) |
|  | Not done | 0 (0%) | 0 (0%) | 3 (.9%) | 3 (.5%) |
| **RUNX3** | Not amplified | 23 (20.4%) | 12 (10.6%) | 80 (23.3%) | 115 (20.2%) |
|  | Amplified | 90 (79.6%) | 101 (89.4%) | 259 (75.5%) | 450 (79.1%) |
|  | Not done | 0 (0%) | 0 (0%) | 4 (1.2%) | 4 (.7%) |

**Supplementary Table 3A:**

**Final logistic regression model coefficients after multiple imputation for Model 1 (neoplasia vs control)**

| **Methylation marker**  **(Sample/Control)** | **N of values imputed** | **Coefficient*** | **95%**  **Confidence Interval** | **P-value** |
| --- | --- | --- | --- | --- |
| **log SFRP2** | 48 | 0.790 | (0.21, 1.37) | 0.008 |
| **log SFRP4** | 36 | 2.690 | (1.63, 3.75) | <0.0005 |
| **log WIF1** | 60 | 0.169 | (-0.27, 0.61) | 0.452 |
| **log APC1A** | 57 | 0.427 | (0.09, 0.76) | 0.013 |
| **log APC2** | 23 | 1.157 | (0.55, 1.76) | <0.0005 |
| **Constant term** | - | -17.742 | (-21.73, -13.75) | <0.0005 |

**coefficients are log odds ratios for a unit increase in log values of the methylation marker. Predictions are made using these coefficient values multiplied by the shrinkage factor of 0.93.*

**Supplementary Table 3B:**

**Final logistic regression model coefficients after multiple imputation for Model 2 (dysplasia vs control)**

| **Methylation marker**  **(Sample/Control)** | **N of values imputed** | **Coefficient*** | **95%**  **Confidence Interval** | **P-value** |
| --- | --- | --- | --- | --- |
| **log SFRP2** | 48 | 1.008 | (0.26, 1.76) | 0.009 |
| **log SFRP4** | 36 | 4.094 | (2.62, 5.57) | <0.001 |
| **log WIF1** | 57 | 0.116 | (-0.41, 0.64) | 0.667 |
| **log APC1A** | 57 | 0.793 | (0.32, 1.27) | 0.001 |
| **log APC2** | 23 | 0.796 | (0.12, 1.47) | 0.021 |
| **Constant term** | - | -22.988 | (-28.79, -17.19) | <0.001 |

**coefficients are log odds ratios for a unit increase in log values of the methylation marker. Predictions are made using these coefficient values multiplied by the shrinkage factor of 0.91.*

**Supplementary Table 3C:**

**Final logistic regression model coefficients after multiple imputation for Model 3 (matched non-neoplastic vs control)**

| **Methylation marker**  **(Sample/Control)** | **N of values imputed** | **Coefficient*** | **95%**  **Confidence Interval** | **P-value** |
| --- | --- | --- | --- | --- |
| **log SFRP4** | 35 | 0.607 | (-0.17, 1.39) | 0.127 |
| **log APC1A** | 57 | 0.464 | (0.15, 0.78) | 0.004 |
| **log SFRP5** | 86 | -0.142 | (-0.40, 0.11) | 0.270 |
| **log SOX7** | 70 | -0.539 | (-0.84, -0.24) | <0.001 |
| **Constant term** | - | -2.540 | (-5.30, 0.22) | 0.071 |

**coefficients are log odds ratios for a unit increase in log values of the methylation marker. Predictions are made using these coefficient values multiplied by the shrinkage factor of 0.90.*

**Supplementary Table 4:**

**Model coefficients and cut-off values for Model 1 neoplastic vs. control analysis after multiple imputation with missing biomarker values**

| **Model** | **log sFRP2** | **log sFRP4** | **log WIF1** | **log APC1A** | **log APC2** | **Constant** | **Cut-off** |
| --- | --- | --- | --- | --- | --- | --- | --- |
| **Full Model** | | | | | | | |
| 1 | 0.790341 | 2.68987 | 0.168881 | 0.426967 | 1.15658 | -17.7416 | 0.40 |
| **Models missing one biomarker** | | | | | | | |
| 2 | 0.9269516 | 3.202865 | 0.4268864 | 0.4359665 |  | -16.95697 | 0.39 |
| 3 | 0.7636128 | 2.746595 | 0.1808004 |  | 1.170222 | -17.57503 | 0.41 |
| 4 | 0.8433857 | 2.710108 |  | 0.4288462 | 1.225026 | -17.71891 | 0.38 |
| 5 | 1.033815 |  | 0.1984314 | 0.4600073 | 1.537024 | -10.08218 | 0.44 |
| 6 |  | 2.926569 | 0.3321497 | 0.400415 | 1.25723 | -17.14115 | 0.42 |
| **Models missing two biomarkers** | | | | | | | |
| 7 | 0.9056502 | 3.251479 | 0.4481147 |  |  | -16.74635 | 0.36 |
| 8 | 1.093859 | 3.344528 |  | 0.4462922 |  | -16.76433 | 0.36 |
| 9 | 0.8201064 | 2.772632 |  |  | 1.244751 | -17.56854 | 0.41 |
| 10 | 1.291991 |  | 0.5715801 | 0.4782231 |  | -6.844074 | 0.38 |
| 11 | 1.019545 |  | 0.227554 |  | 1.561684 | -9.816336 | 0.44 |
| 12 | 1.094246 |  |  | 0.4661336 | 1.626908 | -9.997097 | 0.44 |
| 13 |  | 3.560993 | 0.6468267 | 0.4075435 |  | -16.23202 | 0.40 |
| 14 |  | 2.988017 | 0.3418931 |  | 1.268989 | -17.0741 | 0.42 |
| 15 |  | 3.000233 |  | 0.4011913 | 1.41747 | -17.00814 | 0.41 |
| 16 |  |  | 0.4182801 | 0.4323438 | 1.736938 | -8.405532 | 0.49 |

*Note for the final prediction in module 3 data, these coefficients will be multiplied by the estimated uniform shrinkage factor of 0.93.

**SUPPLEMENTARY FIGURES**

**Supplementary Figure 1: OBF method boundary values**


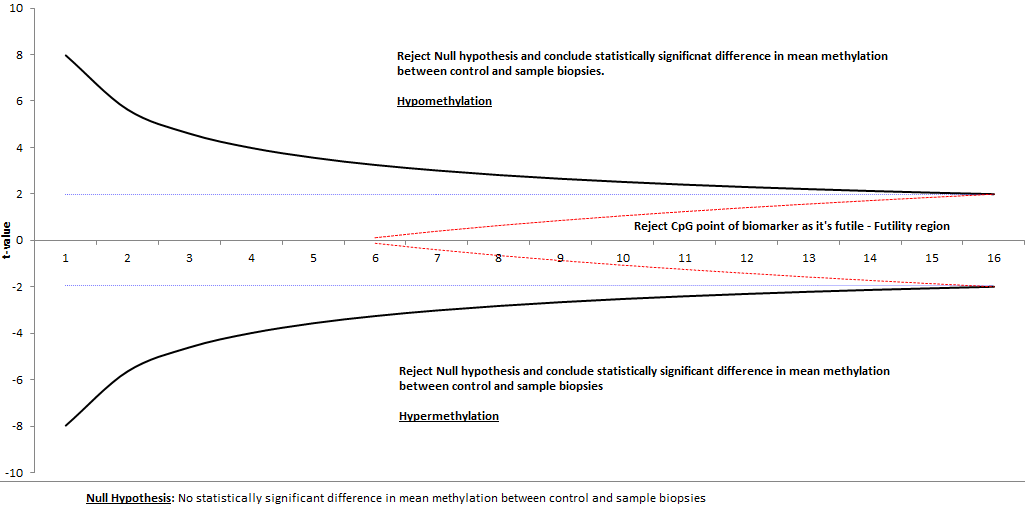


First, results for each batch of samples for each biomarker were evaluated in a group sequential analysis following the O’Brien and Fleming (OBF) method, to assess whether further testing of each biomarker was justified or considered futile. Sequential boundaries were constructed according to the OBF method, the t-statistic computed for each biomarker at each analysis step, and comparison made to the predefined boundary values to test for statistical significance or futility (see figure below). 16 interim analyses were scheduled as we intended to look at the results for each biomarker in cumulative batches of 30 (10 samples and 20 controls). The assays analysed at each batch were mixture of samples and controls to maintain blinding. The t-statistic from the t-test at each analysis was compared to the predefined boundary values to test for significance. Analysis for Neoplastic samples vs Controls and Non-Neoplastic samples vs controls were done separately. X-axis = Analysis batch; Y-axis = t-value

| **Supplementary Figure 2: Box Plots of methylation value per marker for neoplastic, control and matched non-neoplastic mucosa. X-axis = tissue type; Y-axis percentage methylation at methylation marker** | | | |
| --- | --- | --- | --- |
|  |  |  |  |
|  |  |  |  |
|  |  |  |  |

**Supplementary Figure 3:**

**Calibration plot for Model 1 Neoplasia vs Control (after Multiple Imputation). X-axis = Predicted probability; Y-axis = observed probability**

Model 1: Calibration plot

Model 2: Calibration plot

Model 3: Calibration plot
